# Dr. Elisha Townsend

**Published:** 1859-01

**Authors:** 


Obituary.—Died, October 14th, 1858, Dr. Elisha Townsend, in the 54th year of his age.

Perhaps there never was, in the ranks of the dental profession, one more highly respected and beloved, both for his professional ability and social qualities, and whose death has occasioned so much regret, as the subject of this notice. Surely, we never met one in the profession for whom we could entertain more affection, or whose death we could more earnestly mourn and deplore.

In the winter of 1856–'7 he was stricken down by an attack of typhoid fever, from which, after a long illness, he slowly but only partially recovered, yet his earnestness in the cause of dental progress, led him to meet the American Dental Convention, (a creation of his own, and to whom alone belongs all the honor of its origin,) at its session in Boston, in August, 1857. All at that meeting will well remember his cheerful face and many social qualities, and his hearty co-operation in the proceedings of the convention.

In March, of '58, he went to Georgia, also to Florida, to avoid the inclement season at home, and, if possible, to regain his strength, but returned without any material improvement, and in June last, at the suggestion and in the company of a friend, he made a trip to Europe, but got no further than London. While there we received two letters from him, in which, though very hopeful, he wrote, "I am no better yet." The proposition of a united meeting of the American and English dentists in London, as made in our journal, met his hearty concurrence and support, and in furtherance of its accomplishment, he had introduced the subject to many prominent in the profession there, and, consequently, this project occupied the larger part of his letters to us.

After a very short stay in England, during which he received the kindest attentions from the profession there, and finding that instead of gaining he was losing ground, he determined upon a return home, without any such intimation to his friends, and one day, in the latter part of the summer—the day after his arrival—we were astonished at a visit from him, and then, for the first time did we apprehend a serious termination to his sickness—that he had come home to die.

The trip abroad, with its many exposures, and the absence of suitable diet and those little comforts so necessary and grateful to one in his weak condition, had told upon him, greatly emaciating and weakening his system. In but a few days after his return, he was confined to the house, then to his bed, where he lingered and suffered for some two months, when death, so much desired and so patiently waited for, came to his relief, and with all possible peace and calmness his spirit passed away.

Though he rests from his labors, *his works do follow him.*

We had hoped to have been able to give in this number a full and carefully prepared "memoir" of the deceased, but the gentleman in whose hands the matter was placed—an old and intimate acquaintance, and one, than whom there is none more competent for such a work—was unfortunately unable to prepare it in season for us, but gives us the assurance that it shall be in time for our April number.

[*Dental News Letter.*

